# Vesicouterine Fistula (VUF) as a Rare Urogenital Complication Managed with Delayed Surgical Repair: A Case Report and Review of the Literature

**DOI:** 10.1155/2018/2394896

**Published:** 2018-10-24

**Authors:** Evangelos N. Symeonidis, Elias Sdralis, Asterios Symeonidis, Christos Georgiadis, Vasileios Kalyvas, Apostolos Malioris, Michail Papathanasiou

**Affiliations:** ^1^Department of Urology, 424 General Military Hospital of Thessaloniki, Thessaloniki, Greece; ^2^Department of Surgery, 424 General Military Hospital of Thessaloniki, Thessaloniki, Greece

## Abstract

Vesicouterine fistula (VUF) represents a rare urogenital complication. It is considered to be the least common type of urogenital fistulas. Iatrogenic reasons have been shown to be the most prominent cause, with lower segment caesarean section accounting for approximately two-thirds of the cases. The highest incidence concerns young females of reproductive age. VUF can present with clinical symptoms varying from cyclic hematuria, amenorrhea, and vaginal leakage of urine to secondary infertility and first-trimester abortion. Quality of life (QoL) for patients having this pathology is strongly affected due to the psychological burden. Surgical excision of the fistula remains the mainstay of treatment, as less than 5% of patients respond to conservative therapy. Recently laparoscopic and robotic-assisted VUF repair started gaining ground with comparable results to open surgery. Herein, we presented the successful delayed surgical repair of VUF in a 32-year-old female patient. A review of the published literature was also performed, summarizing all the available evidence regarding this rare clinical entity.

## 1. Introduction

Vesicouterine fistula (VUF) is an abnormal communication between the bladder and the uterus. It represents a rare urogenital complication, accounting for approximately 1-4% of genitourinary fistulas [1, 2]. VUF is considered to be the least common type contrary to other types, such as vesicovaginal fistula (VVF) which is the most frequent. It was first described as a clinical entity by A. F. Youssef in 1957 [3].

## 2. Case Report

**Figure 1:**
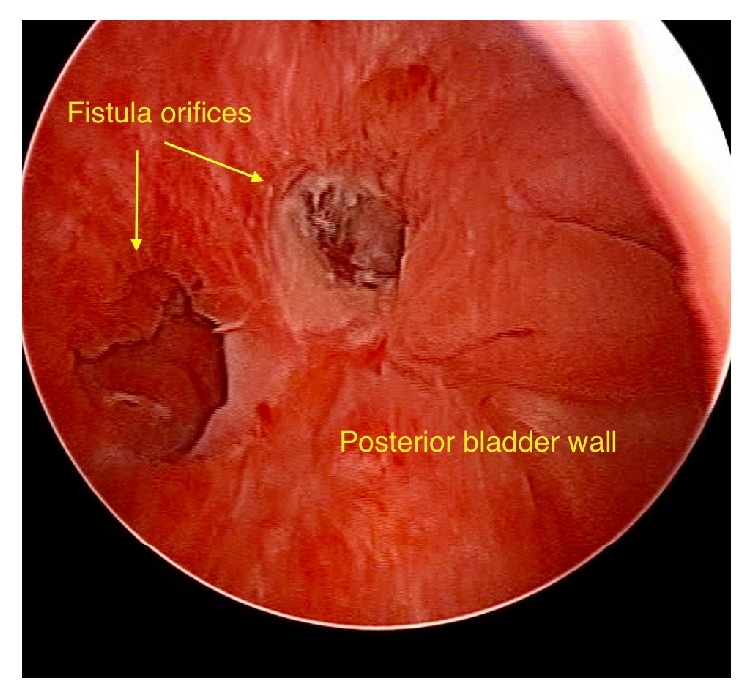
Cystoscopic view of two vesicouterine fistula (VUF) orifices in the posterior bladder wall.

**Figure 2:**
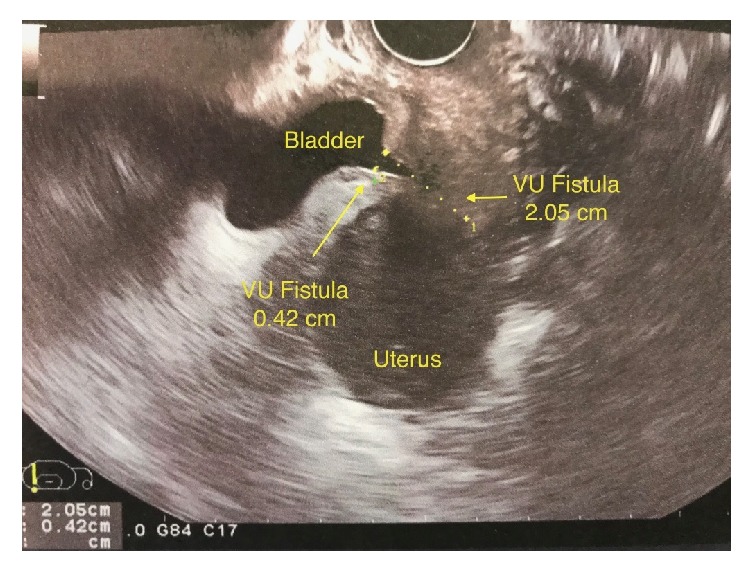
Transvaginal ultrasound revealing the presence of two vesicouterine fistulas.

**Figure 3:**
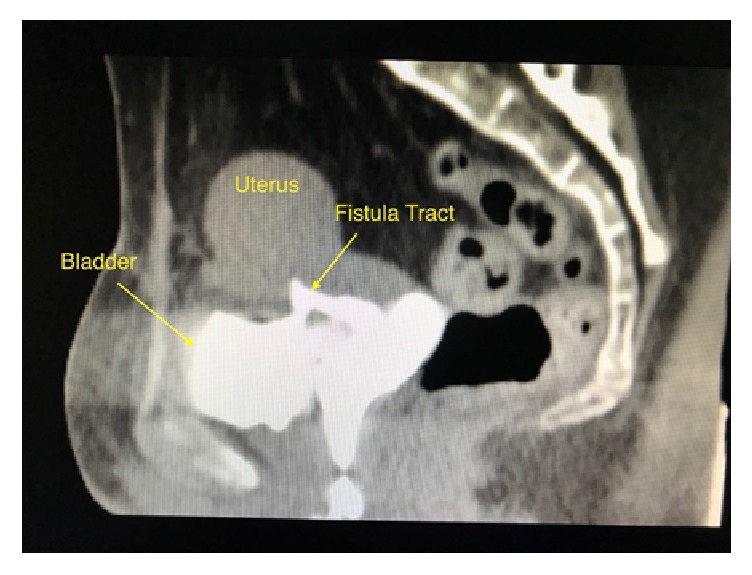
Sagittal view of contrast-enhanced CT scan demonstrating the connection between bladder and uterus.

A 32-year-old female patient was referred to our urology department with intermittent vaginal leakage of urine. According to obstetric history, she underwent a first elective cesarean section in 2014, at 38 weeks of pregnancy. Three years later, despite her will to encounter a vaginal birth after cesarean section (VBAC), at 40 weeks and 3 days of her 2^nd^ pregnancy, it was considered as problematic in association with bladder and uterine rupture, resulting in an emergency C-section. A concurrent restoration of bladder and uterus was performed. One week after her second delivery, the patient noted a watery vaginal discharge. The initial approach was conservative with a 14 French (Fr) Foley catheter draining the bladder for a 2-month period. Meanwhile, she had secondary amenorrhea due to breastfeeding; thus no menstrual bleeding and no cyclic hematuria (menouria) were reported. Her symptoms gradually ameliorated. The 2-month postoperative cystoscopy depicted 2 fistula orifices in the posterior bladder wall (Figure 1). Vaginal ultrasound depicted two fistulas between uterus and bladder (2.05 and 0.42 cm in length) (Figure 2). Moreover, contrast-enhanced computed tomography (CT) scan of the lower abdomen demonstrated the presence of a VUF (Figure 3).

For the next five months, the main symptom was intermittent urine leakage through the vagina, followed by lower urinary tract symptoms (LUTS), due to recurrent infections treated with oral antibiotics.

**Figure 4:**
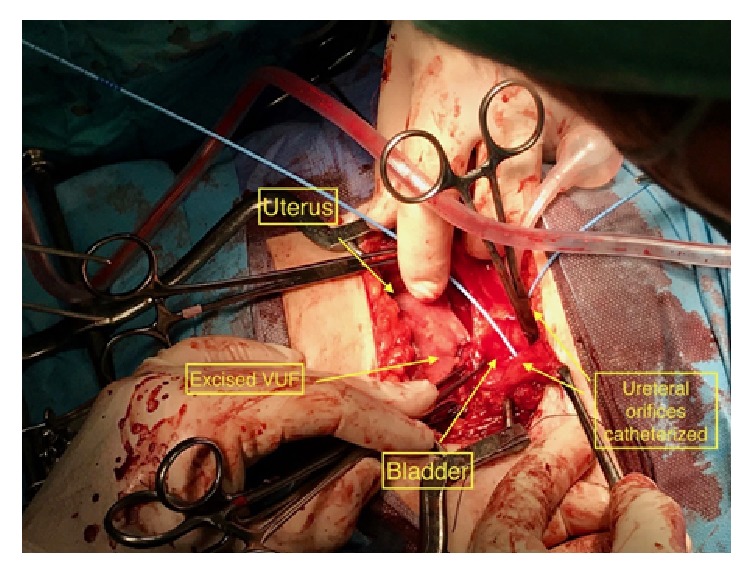
Intraoperative view of uterus, bladder, excised VUF tract, and catheterized ureteral orifices with ureteral stents left for cutaneous externalization.

The VUF was, finally, surgically repaired 7 months after the second emergency caesarean section (C-section). Despite the initial surgical planning for laparoscopic approach, careful preoperative consideration led to the open repair of the VUF. A consensus was reached based on the laborious second delivery, which resulted in a bladder and uterine rupture, as well as the risk for abdominal adhesion development from the previous cesarean sections. Entrance in the abdominal cavity was done through a Pfannenstiel incision. Once the uterus and vesicouterine space were dissected, bladder and uterus were completely detached and the VUF fistula was clearly exposed. A transvesical approach permitted an adequate exposure of the fistula tract. The margin of the fistula was elevated with forceps and excised with scissors circumferentially. The entire fistula tract was dissected. The ureteral orifices were catheterized and the open-ended straight ureteral stents were left aside prior to externalization (Figure 4). The layers of the bladder wall and uterus were adequately delineated, and, after mobilization, they were drawn together with fine sutures without tension. The uterus was closed with 2-0 polyglycolic acid sutures and bladder with 3-0. A 3-way Foley catheter (20 Fr) was inserted into the bladder. Intraoperative anastomosis testing was done with irrigation of 120 ml Methylene Blue (MB) solution mixed with normal saline via the catheter. The assessment of the anastomosis integrity proved to be efficient with no leakage. Both fibrin sealant patch and omental flap were carefully interposed between uterus and bladder. Diligent hemostasis was done and the abdominal wall was closed in layers. Furthermore, drainage was placed in the vesicouterine fold to prevent blood and fluid from draining into the peritoneal cavity. The transverse abdominal incision was closed with running subcuticular suture. The ureteral stents were externalized to a drainage bag on the left (urostomy pouch). Broad-spectrum antibiotics and analgesic regimens were administered for 10 days. There was no need for intravenous opioids.

**Figure 5:**
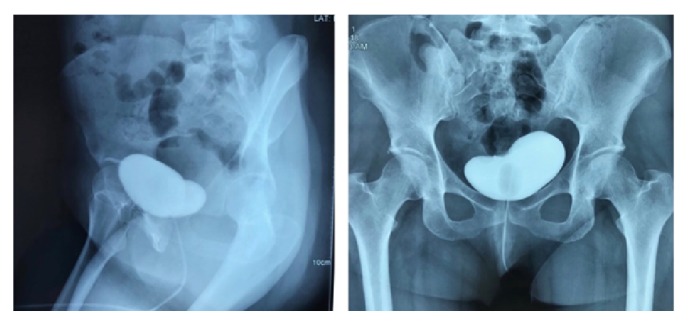
Retrograde cystography image (bladder X-ray) after instillation of iopromide solution (Ultravist®), revealing bladder integrity with no leakage.

Total operative time was 150 min, with no intra- and postoperative complications. Blood loss was less than 100 ml during the first postoperative day. Length of hospital stay (LOS) reached 10 days, with cutaneous externalized ureteral catheters being removed on the 7^th^ postoperative day. Times to oral intake and ambulation were 10 and 17 hours, respectively. The patient made an uneventful postoperative recovery and was discharged from the hospital with a recommendation for follow-up at 3, 6, and 12 months. The indwelling 3-way Foley catheter (20 Fr) left, draining the bladder, for 30 days. Retrograde cystography was given at 1 month, revealing bladder integrity without leakage (Figure 5). Our patient is currently being followed up, with no signs or symptoms of recurrence six months after surgery.

## 3. Discussion

Vesicouterine fistula (VUF) represents a rare urogenital complication. In developed countries, iatrogenic reasons have been shown to be the most common cause, with lower segment cesarean section accounting for approximately two-thirds of the cases [4, 5]. In developing countries, VUF can occur following prolonged and obstructed labor. Other risk factors include manual removal of placenta, abnormal implantation of the placenta, use of forceps during vaginal delivery, previous cesarean sections, uterine rupture, inflammatory bowel disease, and pelvic irradiation [1, 4, 5]. Meanwhile, the highest incidence concerns young females of reproductive age (25-33 years old) [2].

The clinical manifestations vary from cyclic hematuria (menouria), amenorrhea, vaginal leakage of urine, with or without urinary incontinence, and recurrent urinary tract infections associated with low-grade pyrexia to secondary infertility and first-trimester abortion [1, 2, 4–6]. Various imaging procedures have been found useful. Cystoscopy, cystography, and hysterosalpinography (HSG) play a crucial role in the diagnosis of patients with VUF. Additional modalities include contrast-enhanced CT, magnetic resonance imaging (MRI), and transvaginal ultrasound [1, 2, 4–7].

Conservative therapeutic options that have been proposed are bladder catheterization, hormonal therapy, and cystoscopic fulguration of the VUF, all with favorable results. Nevertheless, only 5% of the cases respond to conservative therapy [1, 2, 4–7]. In this way, surgery should be considered the mainstay of treatment in the majority of patients. In addition, many different approaches have been advocated (transperitoneal, transvesical, and transvaginal), along with different surgical techniques of repair (open, conventional laparoscopy, laparoendoscopic single-site surgery, robotic-assisted) [1, 6, 7]. Hysterectomy is warranted in cases of multiparous women around perimenopause and those having uterine pathology [4].

To date, most of the published literature consists of case reports and case-series with a small number of patients and short-term follow-up. The majority of studies have shown satisfactory results in the resolution of VUF. Rajamaheswari N et al. reported 100% success rate in 17 patients who were treated for VUF [4]. Drissi M et al. demonstrated satisfactory functional results in 15 cases of VUF, with an average follow-up of 2.5 years [8]. Similarly, Hadzi‐Djokic JB et al. presented their successful experience in 14 patients and stressed the need for accurate diagnostic evaluation and appropriate use of basic surgical principles [5]. Rao MP et al. managed a relatively young age group of 12 patients with a mean age of 19 years. The surgical outcome was excellent with good continence and resolution of the cyclic hematuria [9]. DiMarco CS et al. reported total resolution of urinary incontinence in all surgically treated patients for VUF [10].

Of note, transvaginal layered repair of VUF seems to be the less preferred approach among surgeons. This may be due to either the complexity of anatomical approach or lack of experience for urological surgeons. It is not until recently that Milani R. et al. managed successfully a VUF in a 43-year-old woman transvaginally. While this approach seems to be safe and effective, they stated that it should only be performed by experienced urogynecological surgeons [1].

Furthermore, it should be noted that the advent of minimally invasive surgery (MIS) has dramatically changed the surgical landscape conferring a number of advantages compared to open surgery. It is well known that both robotics and laparoscopy embody all the principles of minimally invasive techniques, thus providing less postoperative pain, reduced blood loss and risk of transfusion, shorter length of hospital stay, and better cosmesis. Meanwhile, the recent advances in minimally invasive surgery have led to the introduction of laparoendoscopic single-site surgery (LESS), as a less morbid, technically feasible, and efficient alternative to traditional laparoscopy [11]. The largest series of laparoscopic VUF repair has been reported by Abdel Karim et al. In the aforementioned study, 11 females were managed laparoscopically, with 5 of them undergoing extravesical LESS. No complications and no conversion to open surgery were reported. Surprisingly, this represents the first report of LESS repair for VUF in the current body of literature [12]. The analysis of Purkait B et al. comprises the second largest retrospective series of 8 patients with VUF managed with conventional laparoscopy. They conclude that laparoscopic repair is safe, feasible, and effective with successful pregnancy rates in long-term follow-up [7]. According to Maioli et al., surgical success depends on the adherence to good technique rather than the approach. Hence, laparoscopic repair appears to be a viable alternative for surgeons experienced with laparoscopic suturing techniques [13]. In 2009, Hemal AK et al. reported the first worldwide successful case-series of robotic VUF repair in 3 patients, with a mean operative time of 127.5 minutes, average blood loss of 120 ml, and 3 days of hospital stay [14]. Since then, various successful robotic repairs of VUF have been cited [15–17].

Fertility after VUF repair is still a subject of considerable concern. It should, nonetheless, be noted that reported pregnancy rates after VUF surgical repair are encouraging, ranging from 25 to 37.5% [7, 9, 18, 19]. However, our patient was not willing to undergo a third delivery. Moreover, clinicians should be aware of significant impairment in quality of life (QoL) of patients having this pathology. VUF is strongly associated with social and psychological distress [2, 20]. In our case, our patient sustained an unpleasant and altered emotional state till the definitive surgical treatment, strongly connected with the vaginal urinary leakage.

In summary, the adoption of a careful and structured, diagnostic, and operative strategy plays a pivotal role in the definitive treatment of VUF. Surgical management, even with delayed repair, was shown to be feasible, safe, and efficient. Recently, minimally invasive techniques started gaining ground as an alternative approach to traditional open surgical repair, with encouraging and comparable results in the hands of an experienced surgeon. However, there is a need for well-designed studies with a large number of patients and long-term follow-up to support their superiority.
